# Does *Achillea millefolium* extracts possess prokinetic effects on the bovine abomasum thourgh M_3_ muscarinic receptors?

**Published:** 2017-06-15

**Authors:** Mojtaba Mohseni, Masoud Maham, Bahram Dalir-Naghadeh, Ghader Jalilzadeh-Amin

**Affiliations:** 1 *DVSc Candidate, Department of Internal Medicine and Clinical Pathology, Faculty of Veterinary Medicine, Urmia University, Urmia, Iran;*; 2 *Department of Internal Medicine and Clinical Pathology, Faculty of Veterinary Medicine, Urmia University, Urmia, Iran.*

**Keywords:** Abomasal motility, *Achillea millefolium*, Organ bath, Prokinetic

## Abstract

Displacement of the abomasum is a common disease of the gastrointestinal tract in dairy cattle. Abomasal displacement has been associated with abomasal hypomotility. Therefore, it is necessary to identify effective therapeutic agents that stimulate abomasal motility in cattle. Yarrow (*Achillea millefolium*) is traditionally used as a folk remedy for treatment of human gastrointestinal complaints in the northwest of Iran. This study investigated the effects of *A. millefolium* extracts on abomasal smooth muscle preparations from healthy cattle. The *A. millefolium* extracts (3 to3000 mg L^-1^) contracted the isolated of smooth muscle in a concentration-dependent manner, with an effective threshold concentration of 30 mg L^-1^ (*p *< 0.05). The strongest contraction by *A. millefolium* aqueous extracts in a concentration of 3000 mg L^-1 ^was observed and amounted to 124.90 ± 10.80% of the control treatment. This action was unaffected by pretreatment with hexamethonium and indomethacin, but strongly reduced by verapamil, atropine and 4-DAMP. The inhibiting effect of 4-DAMP and atropine suggesting that the effect of *A. millefolium* extracts is mediated at least partly by M_3_ muscarinic acetylcholine receptor. The results suggested that *A. millefolium* has the potential prokinetic effect that may prevent or alleviate dysfunctions of gastrointestinal motility.

## Introduction

Impaired abomasal motility is common in dairy cattle and is suspected to play a major role in the occurrence of left displaced abomasum, abomasal volvulus and abomasal impaction in cattle.^1^^,^^2^ Abomasal motility decreases in many ailments, namely ketosis, hypocalcemia, hyperinsulinemia, and reduced insulin sensitivity that have been studied and reported in recent publications.^3^^-^^7^ Thus, it would be clinically helpful to identify effective prokinetic drugs that stimulate, coordinate and restore abomasal motility in cattle.

The importance of the parasympathetic nervous system in the physiology of GI motility and in the pathophysiology of motility disorders has been described in cattle.^8^ In the cholinergic system, the main endogenous neurotransmitter, acetylcholine (ACh), activates G-protein coupled muscarinic receptors. Smooth muscle contraction in GI organs is the most important effect of the activation of muscarinic receptors located directly on smooth muscle cells or on the nerve cells of the GI nervous system.^9^

Several investigations have been carried out in characterizing muscarinic receptors in the digestive tract of cattle. In the bovine gut smooth muscle, it has been shown that mRNA transcripts and binding sites of M_2_ and M_3_ AChR subtypes are the most abundant with a ratio of 5:1.^10^ In the clinical setting, left-side displacement of the abomasum (LDA) in cows did not alter the extracellular components of M_2_ and M_3_ receptors in the GI tract, whereas receptor densities were lower in the intestinal wall (mainly duodenum) of cows with LDA.^8^

Bethanechol, a well-known prokinetic drug, induces contraction of smooth muscle cells by direct stimulation of muscarinic receptors. *In vitro* studies on smooth muscle preparations revealed a bethanechol-induced, contractility in muscle strips from the esophageal groove of calves^11^ and the abomasum of healthy cows.^12^ However, chemical drugs often have limitations such as serious adverse side effects.^9^

Therefore, herbal products may be an attractive alternative thanks to their lower risk their proved prokinetic effects.^13^^-^^15^ *Achillea millefolium* L. (Yarrow) which belongs to the Asteraceae family is one of the most widely used medicinal plants in the world.^16^

In West Azerbaijan (Iran) the aerial parts of the plant have been used traditionally to treat gastritis, cancer, hemorrhoids, vertigos, anemia, anorexia, dyspepsia, gastralgia, hemorrhage, dysmenorrhea and diarrhea.^17^

Preclinical studies indicate that yarrow may have anti-inflammatory, hepatoprotective, antinociceptive, anxiolytic and antimicrobial activities. Animal studies have also shown that it is generally safe and well tolerated.^16^

In Italy leaves of yarrow are used to make an ointment cooking them over a low heat with olive oil, talon, bee’s wax and egg yolk; this is applied locally to cure sores on bovines’ withers caused by yoke rubbing.^18^ In British Columbia, Canada, the extract of plant flowers has been used to treat diarrhea and gastritis in dogs and cats. It is given orally with a syringe or put in the drinking water.^19^

The main constituents of yarrow are volatile oils (sabinene, β-pinene, 1,8-cineole, artemisia ketone, linalool, α-thujone, β-thujone, camphor, borneol, fenchyl acetate, bornyl acetate, (E)-beta-caryophyllene, germacrene D, caryophyllene oxide, beta-bisabolol, delta-cadinol, chamazulene); flavonoids (apigenin- and luteolin-7-glycosides, and rutin); and alkaloids.^20^

In the current study, we used extracts of yarrow to assess its reported prokinetic action on smooth muscles in the rodent and human gastrointestinal tract,^21^ to investigate their regulatory effects on contractions of the smooth muscles of the bovine abomasum, and also elucidate its mechanism of action.

## Materials and Methods

**Chemicals. **Acetylcholine chloride (ACh), atropine sulfate, hexamethonium, indomethacin, verapamil hydrochloride and 4-DAMP were purchased from Sigma Chemicals Co. (St Louis, USA). Calcium chloride, potassium chloride, sodium chloride, glucose, magnesium sulfate, potassium dihydrogen phosphate, sodium bicarbonate, ethanol and chloroform were obtained from Merck (Darmstadt, Germany). 

**Plant material and extraction. **Aerial parts of *A. millefolium *were collected from the northwest of Iran in 2015. The plant was identified in the Department of Botany, Tarbiat Modarres University of Tehran, Tehran, Iran and a sample was deposited in the herbarium of the Department of Medicinal and Industrial Plants, Urmia University, with the voucher number of 5374. The plant material was cleaned, shade-dried and coarsely grounded. The powdered material was extracted with 70% ethanol by cold maceration for three days with occasional shaking. It was filtered through a muslin cloth and then through a Whatman qualitative grade 1 filter paper (Sigma). 

This procedure was repeated twice and the combined filtrate was evaporated in a rotary evaporator to obtain hydroalcoholic extract of *A. millefolium. *A part of this extract was used in the pharmacological studies and the other part was used for the fractionation. The extract was suspended in distilled water and extracted with chloroform. The mixture was allowed to separate into two layers. The upper layer (aqueous fraction) was again taken into a separating funnel; ethyl acetate was added to it, separated and evaporated with the rotary evaporator to get the ethyl acetate fraction. The remaining lower layer was collected and evaporated to obtain the *A. millefolium* aqueous extract (AMAE).

**Preparation of smooth muscle and data acquisition. **Tissue samples were collected from routinely slaughtered Holstein crossbred dairy cows (4 to 8 years old; n = 18) with no previous history of abomasal displacement or other abomasal disorders. The abomasum was removed within 20 min after stunning. Full-thickness specimens were harvested from the body of the abomasum by dissecting a rectangular piece of tissue (6 × 15 cm) from the location. Tissue specimens were immediately rinsed with cooled (4 ˚C) Krebs solution (composition (mM): NaCl, 118.00; KCl, 4.75; MgSO_4_, 1.20; KH_2_PO_4_, 1.20; CaCl_2_, 2.50; NaHCO_2_, 25.00 and glucose, 11.50). 

Specimens were stored in 1 L of cooled (4 ˚C) Krebs solution that had been oxygenated (95% O_2_ and 5% CO_2_) for 1 hr; and were transported from the slaughterhouse to the laboratory within 15 min. The whole pieces of tissue were placed in a petri dish filled with Krebs solution at room temperature and the mucosa was carefully removed from the muscle layers, and tissue strips (15 × 2 mm) were cut from the abomasal body muscle fibers. 

The abomasal strips were mounted in separated 25 mL chambers, maintained at 37 ˚C in Krebs solution, and gassed continuously with a mixture of 95% O_2_ and 5% CO_2_. One end of each strip was fixed to the bottom of the chamber, and the other end was attached to an isometric muscle transducer (model TRI 202P; PanLab, Barcelona, Spain) coupled to bridge amplifier (model ML224; AD Instruments, Castle Hill, Australia) and data acquisition PowerLab system (model ML870; AD Instruments) using Labchart software (version 8.0, AD instruments). 

Specimens were allowed to equilibrate in the organ bath for 1 hr, whereby muscle tension was preset to 2 g in two steps (1 g each) at 10 min intervals and during this time Krebs solution was replaced every 15 min with fresh solution. All specimens were tested for functional viability prior to and after all experiments by the addition of 1 M acetylcholine to the organ bath. The dose-response curves to determine the effect of acetylcholine were obtained by exposing the preparation to increasing concentrations added to the bath (2 min to each concentration). 

Then the submaximal (inducing responses approximately 70% of the maximum) concentration was determined for each of the strips. Strips producing three consistent repeatable responses to submaximal concentration of acetylcholine were used. 

After removal of acetylcholine through wash out, the abomasum responses were observed in the presence of increasing cumulative concentrations of *A. millefolium *aqueous and hydroalcoholic extracts (3 to 3000 mg L^-1^).

To assess the possible mechanisms underlying the contractile effect of the aqueous fraction on abomasal strips, atropine, a muscarinic receptor blocker (10 µM); hexamethonium, a nicotinic nACh (NN) receptor anta-gonist (10 µM); indomethacin, a prostaglandin synthesis inhibitor (10 µM); and 4-DAMP, a muscarinic M_3_ receptor antagonist (10 µM) and verapamil, a calcium channel blocker (0.1 µM) was added to the organ bath 10 min prior to the addition of the extract (3 to 3000 mg L^-1^).

**Statistical analysis. **Data were examined graphically for assumptions of normal distribution and homogeneity of variation. Nonparametric statistical test was used for analysis because the assumptions were not met normal distribution. The Friedman repeated measures analysis of variance on ranks was used to compare results in strength of contractions between different concentrations of the extract. Overall *p *< 0.05 was considered significant. Pair-wise comparisons between each treatment group (concentration) versus control group were made using Dunnett's Method. 

Results are expressed as medians and interquartile ranges (25^th^ - 75^th^ percentiles). Data were analyzed using SigmaPlot for windows (version 12.3; Systat Software, Inc. San Jose, USA).

## Results

We tested 72 specimens by the use of Acetylcholine chloride to determine functional viability and only six specimens did not respond to stimulation with Acetylcholine chloride. All 66 specimens tested at the end of the recording period had the expected response to Acetylcholine chloride and showing that the muscle was not damaged by non-specific action. 

Acetylcholine (1 to 10 µM) caused a concentration-dependent contraction of the cattle abomasum. 

The solvent as a control (distilled water), did not exert an effect on basal tonus of any preparations of cattle abomasum. 

**Fig. 1.**
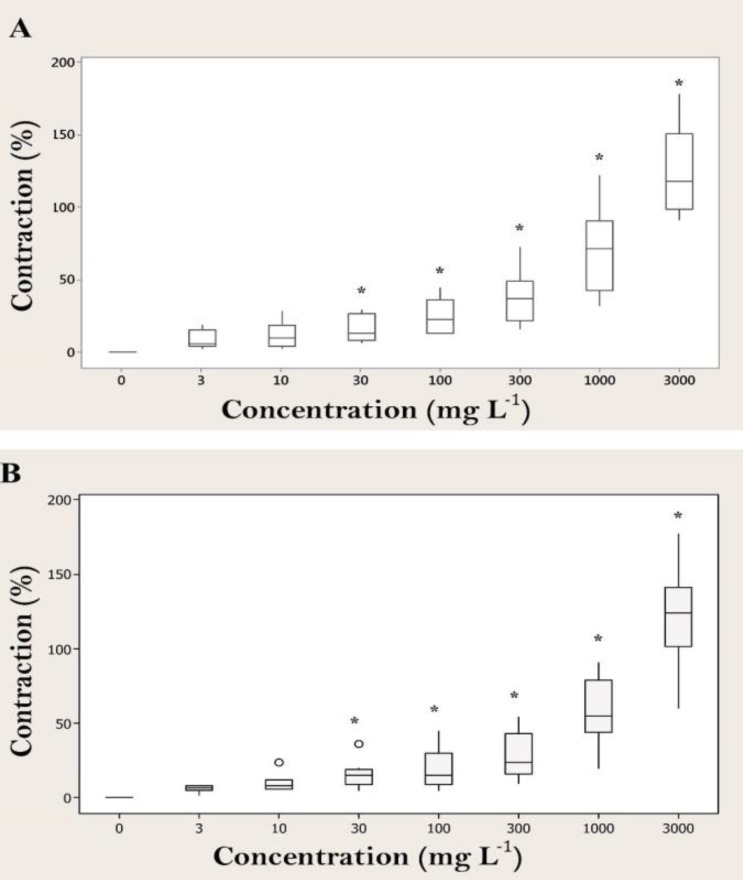
Box plots for effects of *A. millefolium *aqueous extract (n = 8) (**A**) and hydroalcoholic extract (n = 8) (**B**) on basal tonus of healthy cattle abomasal preparations. Each box represents the central 50% of the values, the horizontal line within each box represents the median value, and the whiskers indicate the range of values that are within the inner boundary. Values outside the inner fence are plotted as empty circles (o).

**Fig. 2:**
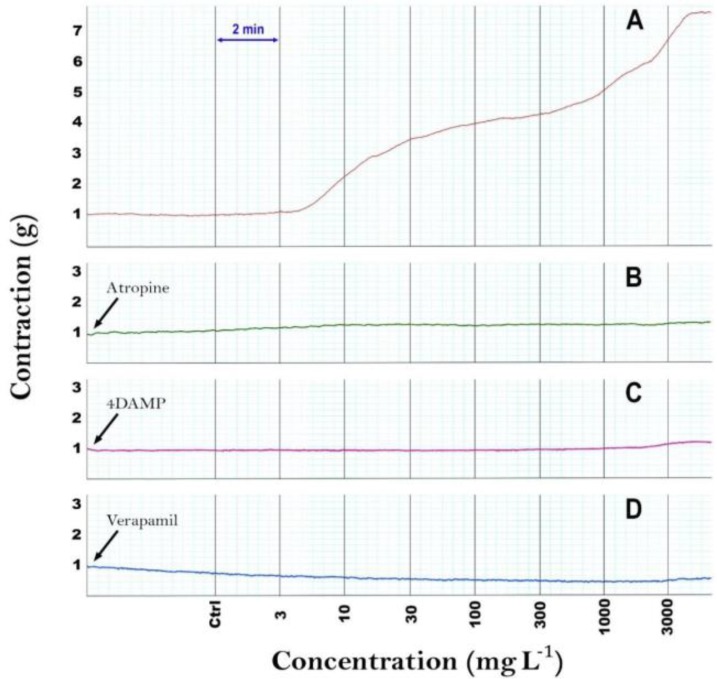
Basal contractions and dose dependent response of abomasal smooth muscle to AMAE (**A**). Inhibitory effects of atropine (**B**), 4-DAMP (**C**), and verapamil (**D**) on the AMAE-induced contractions of abomasal smooth muscle

The *A. millefolium *aqueous and hydroalcoholic extracts (3 to 3000 mg L^-1^) contracted the isolated strips of cattle abomasum smooth muscle in a concentration-dependent manner, with an effective threshold concentration of 30 mg L^-1^ (*p *< 0.05, Fig. 1) but treatment of tissue strips with 3 and 10 mg L^-1^ of *A. millefolium* extracts did not show any significant difference with the control. A typical trace, showing the effect of the *A. millefolium *aqueous extract (3 to 3000 mg L^-1^) is covered in Figure 2A.

When the organ bath was drained and Krebs was added, there was a rapid relaxation of the muscle to baseline of resting tension. Atropine at 10 µM, concentration did not affect the spontaneous smooth muscle contractions but abolished the contractile effect of Ach. Treatment of the tissues with atropine (10 µM) completely abolished AMAE induced smooth muscle contractions (Fig. 2B). Furthermore, the M3 muscarinic receptor antagonist 4-DAMP (10 µM) revealed an inhibition of contractions caused by AMAE (Fig. 2C), while either hexamethonium (10 µM) or indomethacin (10 µM) had no impact (data not shown).

Verapamil at the bath concentration of 0.1 µM dramatically inhibited the contractile amplitude and tension induced by AMAE (Fig. 2D).

٭ indicates significant differences compared to control (*p *< 0.05).

## Discussion

The main findings of this study were that the *A. millefolium* extracts induced a significant increase in contractility of smooth muscle preparations of abomasum. The spasmogenic effect of aqueous extract was completely abolished in the tissues pretreated with atropine, similar to that of ACh. Results of this study confirmed the presence of cholinergic constituent(s) in the extract of the plant responsible for the spasmogenic response. Acetylcholine ,a neurotransmitter of the parasympathetic nervous system, has an important role in abomasal motility,^22^ and its action is mediated by the activation of muscarinic receptors, whereas atropine is a muscarinic receptor blocker.^12^

Our findings were in agreement with a previous study that demonstrated the aqueous extract of dried flower heads of yarrow exerted a direct spasmogenic effect on mouse and human gastric antrum.^21^

When the stimulant effect of AMAE was studied in the presence of hexamethonium, a ganglion blocker or indomethacin, a prostaglandin synthesis inhibitor, it remained unchanged, suggesting the presence of ACh-like constituents independent of nicotine receptors activation or prostaglandin synthesis inhibition. 

Acetylcholine, the main endogenous neurotransmitter in the cholinergic system, causes a contraction of the smooth muscle layers in the forestomach and abomasum through activation of muscarinic receptors^8^^,^^23^ located directly on smooth muscle cells or nerve cells of enteric nervous system.^24^ It has been demonstrated that M_3_ mAChRs play a predominant role in the mediation of contraction in smooth muscle preparations, even though M_2_ mAChRs were detected at higher density than M_3_ mAChRs.^25^^,^^26^

Regarding in these information, we examined whether AMAE-induced contraction was mediated, at least, by activation of the M_3 _muscarinic receptors and observed that the muscarinic M_3_ receptor-preferring antagonist, 4-DAMP, completely blocked the AMAE-induced contractions.

Although it has been suggested that the extent of contribution of the muscarinic M_3_ receptors differs with the type of smooth muscles and the species of animals,^27^ we consider that AMAE may have high affinity with the muscarinic M_3_ receptors, Accordingly the contractile response of the abomasal smooth muscle to AMAE may chiefly result from the activation of muscarinic M_3_ receptors. However, to clearly understand these speculations, further studies are needed to elucidate the pharmacological affinity of AMAE with the muscarinic receptor subtypes.

Pretreatment of tissues with verapamil, a standard calcium channel blocker, inhibited muscle contractions induced by AMAE, confirming the action of extract on abomasal motility was medicated by Ca^2+^ influx. The M_3_ muscarinic receptor is coupled to Gq-type G proteins, resulting in the activation of the second messengers inositol trisphosphate which induces Ca^2+^ release from intracellular Ca^2+^.^28^

In smooth muscle cells, increase in cytoplasmic calcium concentration is the primary stimulus for contraction, which is generally the result of both release of intracellular stored calcium and the influx of extracellular calcium.^29^

The main pathway for Ca^2+^ entry into intestinal smooth muscle cells is through L-type Ca^2+^ channels. Verapamil is a calcium channel blocker that has been used to promote the relaxation of smooth muscle cells by inhibiting calcium influx through calcium channels and calcium release from intracellular stores.^30^^,^^31^

In a previous study, the chemical composition of yarrow has been analyzed in detail and extracts of this plant have been demonstrated to contain a number of pharmacological active ingredients, including alkaloids such as choline and flavonoids such as rutin and apigenin.^21^ Borrelli *et al*. also indicated that choline, is the chemical ingredient of yarrow responsible for the gastric contractile effect. Further studies are warranted to investigate the *in vivo* effects of AMAE in animals suffering from LDA.
